# Risk and Factors associated with disease manifestations in systemic lupus erythematosus – lupus nephritis (RIFLE-LN): a ten-year risk prediction strategy derived from a cohort of 1652 patients

**DOI:** 10.3389/fimmu.2023.1200732

**Published:** 2023-06-15

**Authors:** Shirley C. W. Chan, Yong-Fei Wang, Desmond Y. H. Yap, Tak Mao Chan, Yu Lung Lau, Pamela P. W. Lee, Wai Ming Lai, Shirley K. Y. Ying, Niko K. C. Tse, Alexander M. H. Leung, Chi Chiu Mok, Ka Lai Lee, Teresa W. L. Li, Helen H. L. Tsang, Winnie W. Y. Yeung, Carmen T. K. Ho, Raymond W. S. Wong, Wanling Yang, Chak Sing Lau, Philip H. Li

**Affiliations:** ^1^ Division of Rheumatology & Clinical Immunology, Department of Medicine, Queen Mary Hospital, The University of Hong Kong, Hong Kong, Hong Kong SAR, China; ^2^ Department of Paediatrics & Adolescent Medicine, Queen Mary Hospital, The University of Hong Kong, Hong Kong, Hong Kong SAR, China; ^3^ School of Life and Health Sciences, School of Medicine and Warshel Institute for Computational Biology, The Chinese University of Hong Kong, Shenzhen, Guangdong, China; ^4^ Division of Nephrology, Department of Medicine, Queen Mary Hospital, The University of Hong Kong, Hong Kong, Hong Kong SAR, China; ^5^ Department of Paediatrics & Adolescent Medicine, Princess Margaret Hospital, Hong Kong, Hong Kong SAR, China; ^6^ Department of Medicine & Geriatrics, Princess Margaret Hospital, Hong Kong, Hong Kong SAR, China; ^7^ Department of Medicine, Queen Elizabeth Hospital, Hong Kong, Hong Kong SAR, China; ^8^ Department of Medicine, Tuen Mun Hospital, Hong Kong, Hong Kong SAR, China; ^9^ Department of Medicine, Pamela Youde Nethersole Eastern Hospital, Hong Kong, Hong Kong SAR, China

**Keywords:** systemic lupus erythematosus, lupus nephritis, risk assessment, prediction, model

## Abstract

**Objectives:**

Lupus nephritis (LN) remains one of the most severe manifestations in patients with systemic lupus erythematosus (SLE). Onset and overall LN risk among SLE patients remains considerably difficult to predict. Utilizing a territory-wide longitudinal cohort of over 10 years serial follow-up data, we developed and validated a risk stratification strategy to predict LN risk among Chinese SLE patients – Risk and Factors associated with disease manifestations in systemic Lupus Erythematosus – Lupus Nephritis (RIFLE-LN).

**Methods:**

Demographic and longitudinal data including autoantibody profiles, clinical manifestations, major organ involvement, LN biopsy results and outcomes were documented. Association analysis was performed to identify factors associated with LN. Regression modelling was used to develop a prediction model for 10-year risk of LN and thereafter validated.

**Results:**

A total of 1652 patients were recruited: 1382 patients were assigned for training and validation of the RIFLE-LN model; while 270 were assigned for testing. The median follow-up duration was 21 years. In the training and validation cohort, 845 (61%) of SLE patients developed LN. Cox regression and log rank test showed significant positive association between male sex, age of SLE onset and anti-dsDNA positivity. These factors were thereafter used to develop RIFLE-LN. The algorithm was tested in 270 independent patients and showed good performance (AUC = 0·70).

**Conclusion:**

By using male sex, anti-dsDNA positivity, age of SLE onset and SLE duration; RIFLE-LN can predict LN among Chinese SLE patients with good performance. We advocate its potential utility in guiding clinical management and disease monitoring. Further validation studies in independent cohorts are required.

## Introduction

Systemic lupus erythematosus (SLE) is a complex autoimmune disease with a wide range of heterogenous manifestations. The clinical diagnosis of SLE is usually made in reference to the various versions of classification criteria (1–3). Nonetheless a fundamental disadvantage of using such systems is that individuals who fulfil such criteria for the umbrella diagnosis of “SLE” can vary hugely and there remains much patient heterogeneity. Individuals can vary widely in terms of different organ involvement, disease severity and/or laboratory abnormalities. This diverse heterogeneity also leads to huge diagnostic and therapeutic challenges, imposing limitations on disease diagnosis, prognostication, and treatment (4–6).

A prime example of such heterogeneity is lupus nephritis (LN), with its incidence and prevalence varying greatly across different populations and ethnicities. LN remains one of the most severe organ manifestations, and affects up to 50-60% of SLE patients (5, 7, 8). Despite advancement in treatment strategies, the risk of progression to end-stage renal disease (ESRD) remained at 10-30% (9). The prevalence and incidence of LN varies greatly depending on a myriad of factors, including age of disease onset, sex, ethnicity, and autoantibody profile (4, 10–12). However, studies investigating the incidence of LN remain scarce, and longitudinal studies investigating risk factors associated with LN development are lacking. Identification of patients at risk of LN development will facilitate early diagnosis and treatment to minimize risk of organ damage.

Prediction models provide the foundation for personalized medicine and risk prediction models have been increasingly utilized to assist clinical decision making (13, 14). Given the diverse heterogeneity in SLE patients, prediction models may potentially be useful to inform physicians and patients on possible disease manifestations and outcomes (15). For example, patients deemed at higher risk of developing LN may benefit from more intensive or frequent monitoring, have lower threshold for decision towards invasive renal biopsies or costly immunosuppressive therapies.

To tackle the unpredictability of SLE manifestations and enhanced personalized medicine for SLE patients, we recruited a territory-wide longitudinal cohort to develop prediction strategies for disease manifestations and flares – Risk and Factors associated with disease manifestations in systemic Lupus Erythematosus (RIFLE). Only patients with at least 10 years of serial follow-up data were recruited. In this study involving a multidisciplinary team of bioinformaticians, immunologists, paediatricians, nephrologists and rheumatologists, we developed and validated a risk stratification strategy to predict LN risk among Chinese SLE patients - RIFLE-LN.

## Methods

RIFLE-LN was established with patient recruitment from eight major tertiary referral centres across Hong Kong. Only physician diagnosed SLE patients with at least 10 years of serial follow-up data or all-cause deaths were included and longitudinally analysed. Patients were regularly followed-up according to their clinical need (with average frequency of around every 4 months). Patients of Chinese descent were included (reported by patients). Clinical, blood and urine results were documented during each visit. Data from all clinical notes and visits were extracted for analysis; this included age of disease onset, gender, presence of SLE-related autoantibodies (ever-positive), urine results, clinical manifestations, renal biopsy results. All patients with a physician diagnosis of LN based on clinical and laboratory evidence suggestive of disease activity and renal involvement were identified. This included the presence of persistent proteinuria (urine protein concentration >0·5 gram per day on ≥ 2 occasions), urinary cellular casts, or histological evidence of LN (2).

In patients with biopsy-proven LN, histological classes according to the International Society of Nephrology/Renal Pathology Society classification were recorded (16). Estimated glomerular filtration rate (eGFR) was calculated using modification of diet in real disease (MDRD) formula. Chronic kidney disease (CKD) was defined according to the Kidney Disease: Improving Global Outcome classification as eGFR<60ml/min/1·73m^2^ for three months or more (17). End-stage renal disease (ESRD) was defined as stage 5 chronic kidney disease (eGFR <15ml/min/1·73m^2^) or the initiation of long-term renal replacement therapy (RRT).

Patients were randomly assigned to either the training and validation cohort and the testing cohort in 5:1 ratio. Based on clinical and serological features in the training and validation cohort, a prediction model was developed based on factors that showed statistically significant association with LN in regression analysis and SLE duration. Generalized linear model (GLM) was used to predict the probability of LN based on age of SLE onset, male sex, and anti-dsDNA positivity. The results from GLM were used to generate the predicted probability of LN at various timepoints after SLE onset. Data from 90% of patients were randomly selected for training and the remaining 10% were used for validation. The process was repeated 10 times to overcome selection bias. The values with greatest sensitivity and specificity after ten cycles of training and validation were selected.

### Statistical analysis

Continuous variables were expressed as median with interquartile range (IQR) and compared using Mann-Whitney U test. Categorical variables were expressed as percentage and compared using chi-square test. Cox regression analysis was performed to identify variables associated with LN. Variables with p-value <0·1 in the univariate analysis were included in the multivariate model. Variables with p-value <0·05 in multivariate analyses were considered as significant. Hazard ratios were reported with 95% confidence interval. The variables were tested in using log rank test.

Prediction performance of RIFLE-LN was evaluated in the testing cohort using area under the receiver operating curve (AUC-ROC). The performance was defined as excellent (AUC 0·9-1), very good (AUC 0·8-0·9), good (AUC 0·7-0·8), satisfactory (AUC 0·6-0·7) or unsatisfactory (0·5-0·6). *R version 4·0·3* was used for the development of the prediction model, and *SPSS Statistics version 28* was used for other statistical analyses.

## Results

A total of 1652 Chinese patients with SLE were recruited with a median age of disease onset at 29 years. The median duration of follow-up was 21 years. 1382 patients were assigned for training and validation of the RIFLE-LN model, while 270 were assigned for testing. All patients were tested positive for anti-nuclear antibody. Details of patient demographics, disease manifestations, autoantibody profiles, major organ involvement, mortality and breakdown of various renal profiles for LN patients in the training and validation cohort and the testing cohort are shown in **Tables 1**, **2**, respectively. Patient characteristics of the two cohorts were comparable, except a higher frequency of anti-Sm and anti-La in the testing cohort (**Supplementary Table 1**). Use of immunosuppressive agents is summarised in **Table 3**.

**Table 1:** Clinical and serological features of training and validation cohort.

	All SLE (N=1382)	LN (N=845)	Never LN (N=537)	OR (95% CI)	p-value
**Age of SLE onset (median, IQR)**	29 (18)	26 (17)	33 (18)	––––	<0.001
<18 years	252/1382 (18.2%)	195/845 (23.1%)	57/537 (10.6%)	2.53 (1.84-3.47)	<0.001
18-50 years	1035/1382 (74.9%)	603/845 (71.4%)	432/537 (80.4%)	0.61 (0.50-.079)	<0.001
>50 years	95/1382 (6.9%)	47/845 (5.6%)	48/537 (8.9%)	0.60 (0.40-0.91)	0.016
**Male sex**	116/1382 (8.4%)	86/845 (10.2%)	30/537 (5.6%)	1.92 (1.25-2.95)	0.003
**Duration of follow-up (median, IQR)**	21 (11)	21 (11)	19 (10)	––––	<0.001
Auto-antibodies					
Anti-dsDNA	1096/1382 (79.3%)	713/845 (84.4%)	383/537 (71.3%)	2.17 (1.67-2.83)	<0.001
Anti-Ro	420/898 (46.8%)	241/532 (45.3%)	179/366 (48.9%)	0.87 (0.66-1.13)	0.288
Anti-RNP	201/898 (22.4%)	117/532 (22.0%)	84/366 (23.0%)	0.95 (0.69-1.30)	0.735
Anti-La	110/898 (12.2%)	60/532 (11.3%)	50/366 (13.7%)	0.80 (0.54-1.20)	0.284
Anti-Sm	94/898 (10.5%)	49/532 (9.2%)	45/366 (12.3%)	0.73 (0.47-1.11)	0.138
Anti-phospholipid	262/1146 (22.9%)				
Major organ involvement					
Hematological	805/1382 (58.2%)	512/845 (60.6%)	293/537 (54.65)	1.28 (1.03-1.59)	0.027
Neuropsychiatric	187/1382 (13.5%)	120/845 (14.2%)	67/537 (12.5%)	1.16 (0.84-1.60)	0.361
■ Seizure disorders	45/187				
■ Cerebrovascular disease	43/187				
■ Mononeuropathy/polyneuropathy	19/187				
■ Others (acute confusion, aseptic meningitis, cognitive dysfunction, demyelinating syndrome, mood disorder, myasthenia graves, myelopathy, psychosis)	80/187				
Pulmonary	150/1382 (10.9%)	81/845 (9.6%)	69/537 (12.8%)	0.72 (0.51-1.01)	0.057
■ Interstitial lung disease	68/150				
■ Pleural effusion/pleurisy	50/150				
■ Pulmonary hypertension	29/150				
■ Others	3/150				
Cardiac	64/1382 (4.6%)	42/845 (5.0%)	22/537 (4.1%)	1.22 (0.72-2.08)	0.451
■ Pericardial effusion/pericarditis	31/64				
■ Myocarditis	30/64				
■ Libman-sacks endocarditis	3/64				
Gastrointestinal	59/1382 (4.3%)	34/845 (4.0%)	25/537 (4.7%)	0.86 (0.51-1.46)	0.571
■ Protein losing enteropathy	47/59				
■ Mesenteric vasculitis	8/59				
■ Pseudo-obstruction	2/59				
■ Others	2/59				
Lupus nephritis#					
Biopsy-proven		706/845 (83.6%)			
■ Class III (+/- V)		161/845			
■ Class IV (+/- V)		376/845			
■ Class V		110/845			
■ Others (Class I or Class II +/- V)		59/845			
Renal outcomes					
End-stage renal failure	74/1382 (5.4%)	67/845 (7.9%)	7/537 (1.3%)	6.52 (2.97-14.31)	<0.001
Renal replacement therapy	53/1382 (3.8%)	52/845 (6.2%)	1/537 (0.2%)	35.1 (4.8-255.0)	<0.001
**Death**	139/1382 (10.1%)	93/845 (11.0%)	46/537 (8.6%)	1.32 (0.91-1.91)	0.142
Age of death (median, IQR)	57 (24)	56 (22)	59 (17)	––––	0.093

CI, confidence interval; IQR, interquartile range; OR, odds ratio; SLE, systemic lupus erythematosus; LN, lupus nephritis.

# Lupus nephritis was diagnosed based on clinical and laboratory findings (persistent proteinuria >0.5g per day, urinary cellular casts, or histological evidence of LN) suggestive of disease activity and renal involvement.

**Table 2:** Clinical and serological features of testing cohort.

	N=270
**Age of SLE onset (median, IQR)**	30 (15)
<18	30/270 (11.1%)
18-50	215/270 (79.6%)
>50	25/270 (9.3%)
**Male sex**	27/270 (10.0%)
**Duration of follow-up (median, IQR)**	19 (15)
Auto-antibodies	
Anti-dsDNA	218/270 (80.7%)
Anti-Ro	98/195 (50.3%)
Anti-RNP	49/195 (25.1%)
Anti-La	35/195 (17.9%)
Anti-Sm	33/195 (16.9%)
Anti-phospholipid	38/151 (25.2%)
Major organ involvement	
Haematological	165/270 (61.1%)
Neuropsychiatric	25/270 (9.3%)
■ Seizure disorders	6/25
■ Cerebrovascular disease	4/25
■ Mononeuropathy/polyneuropathy	4/25
■ Others (acute confusion, demyelinating syndrome, mood disorder, myasthenia graves, myelopathy, psychosis)	11/25
Pulmonary	35/270 (13.0%)
■ Pleural effusion/pleuritis	12/35
■ Interstitial lung disease	10/35
■ Pulmonary hypertension	11/35
■ Others	2/35
Cardiac	11/270 (4.1%)
■ Pericardial effusion/pericarditis	8/11
■ Myocarditis	3/11
Gastrointestinal	8/270 (3.0%)
■ Protein losing enteropathy	5/8
■ Mesenteric vasculitis	2/8
■ Pseudo-obstruction	1/8
Lupus nephritis#	148/270 (54.8%)
■ Class III (+/- V)	24/148
■ Class IV (+/- V)	64/148
■ Class V	27/148
■ Others (Class I or Class II +/- V)	10/148
■ No renal biopsy	23/148
Renal outcomes	
End-stage renal failure	10/270 (3.7%)
Renal replacement therapy	9/270 (3.3%)
**Death**	27/270 (10.0%)
Age of death (median, IQR)	59 (13)

CI, confidence interval; IQR, interquartile range; OR, odds ratio; SLE, systemic lupus erythematosus.

# Lupus nephritis was diagnosed based on clinical and laboratory findings (persistent proteinuria >0.5g per day, urinary cellular casts, or histological evidence of LN) suggestive of disease activity and renal involvement.

**Table 3:** Use of immunosuppressive agents among 1652 patients with SLE.

Immunosuppressive agents	Number of patients (%)
Prednisolone	1572 (95.2%)
Hydroxychloroquine	1164 (70.5%)
Azathioprine	943 (57.1%)
Mycophenolic acid	674 (40.8%)
Cyclophosphamide	350 (21.2%)
Cyclosporin	128 (7.7%)
Tacrolimus	97 (5.9%)
Rituximab	36 (2.2%)
Belimumab	21 (1.3%)

### Burden of LN among Chinese patients with SLE was significant, especially in early disease course

**Figure 1:**
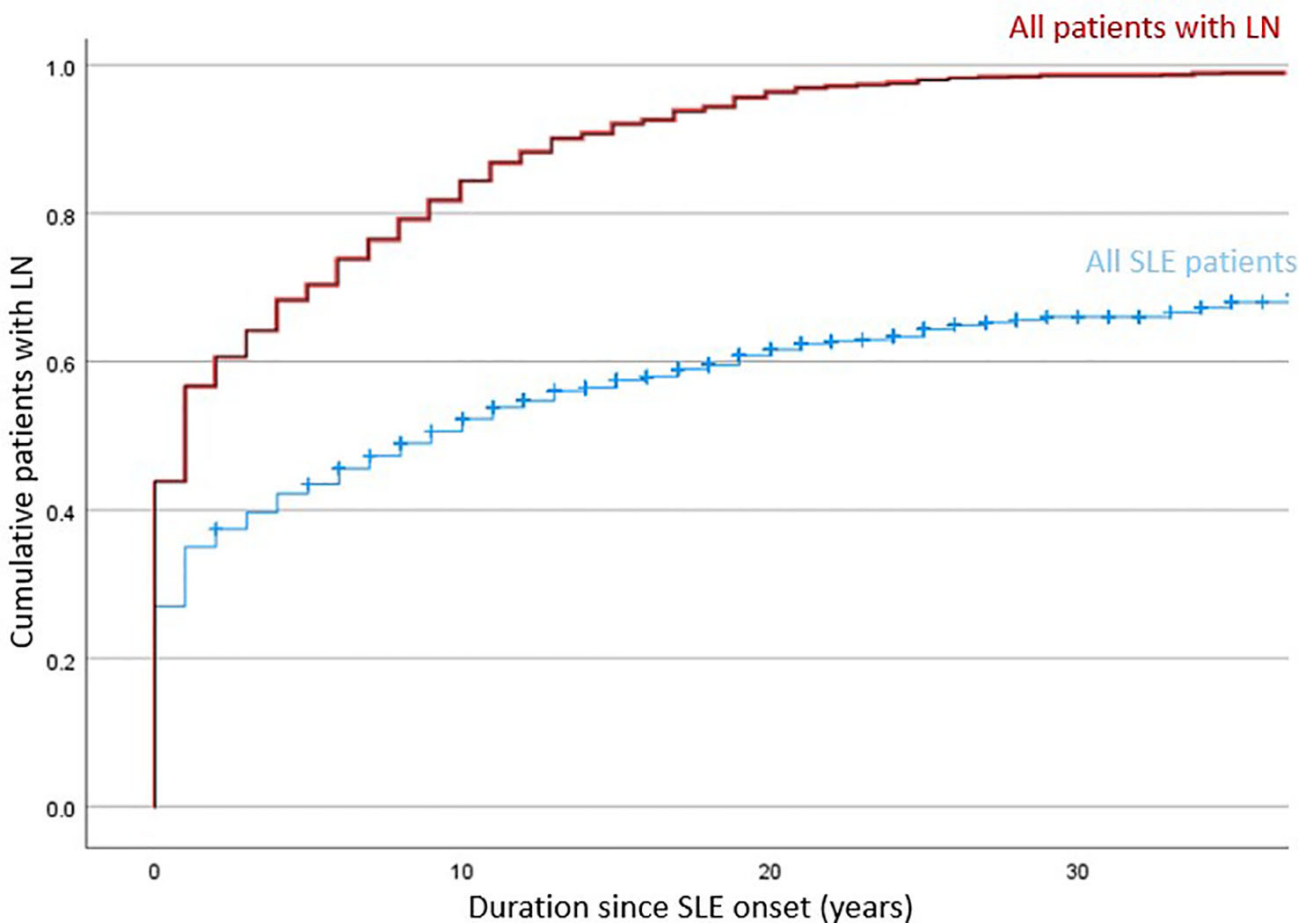
Incidence of LN among 1382 patients in the training and validation cohort of RIFLE-LN.

The training and validation cohort included 1382 patients with SLE, and lupus nephritis developed in 845 (61·1%) patients primarily manifested early in the disease course (**Figure 1**). Among patients who ever developed LN, 45·4%, 71·1% and 85·2% presented within their first year, 5 years and 10 years of SLE onset; respectively. A total of 707 (83·7%) patients had biopsy-proven LN. Proliferative (or mixed proliferative and membranous) LN were the most common histological subtypes and occurred in 537 (63·6%) patients. Pure membranous LN occurred in 111 (13·1%) patients. ESRD developed in 67 (7.9%) of LN patients after a median of 16·5 years after LN onset. A total of 139 deaths were observed with a median age of death of 57 years. Infection, cardiovascular events, and malignancy were the most common causes of death. Other causes included active SLE, pancreatitis, suicide, and surgical complications. There was no significance difference in mortality and age of death in patients who developed LN.

### Young age of SLE onset, male sex and anti-dsDNA autoantibody were associated with LN development

**Figure 2:**
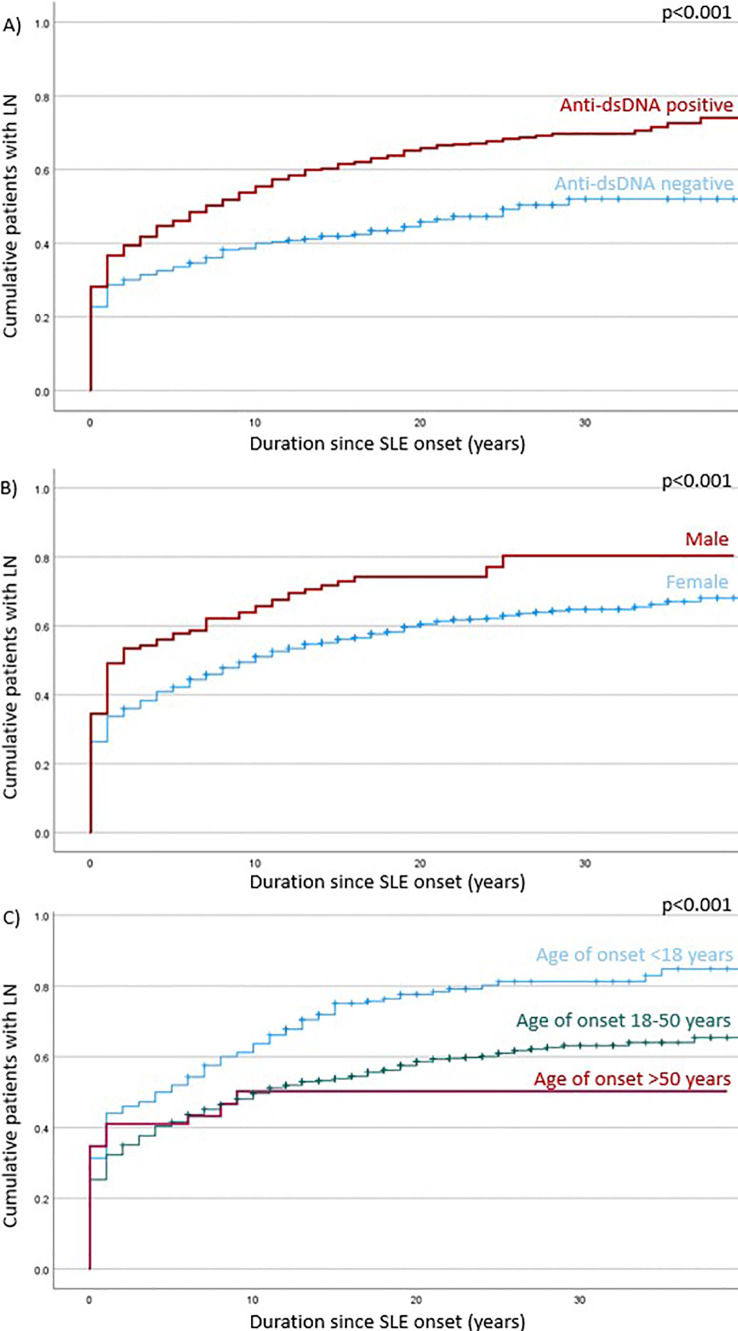
Log rank test of factors associated with LN development: **(A)** – antids-DNA positivity; **(B)** – male sex; **(C)** – age of SLE onset.

Variables including age of SLE onset, male sex, autoantibodies and clinical features were evaluated for their association with LN in Cox regression analysis (**Table 4**). In univariate analysis, factors associated with LN included young age of onset, male sex, anti-dsDNA, and haematological involvement; whereas pulmonary involvement was a negative predictor. Multivariate analysis showed that young age of onset (HR 1.50, 95% CI 1.09-2.06, p=0.013), male sex (HR 1·40, 95% CI 1·12-1·75, p=0·003), and anti-dsDNA autoantibody positivity (HR 1·57, 95% CI 1·30-1·90, p<0·001) were significantly associated with LN; pulmonary involvement remained a negative predictor (HR 0.78, 95% CI 0.62-0.98, p=0.035). Considering the limited number of patients with pulmonary involvement in the training and validation cohort (accounting for 10.9%), only young age of onset, male sex, anti-dsDNA autoantibody and disease duration were further used for model development. **Figure 2** represents the Kaplan-Meier analysis showing the effects of anti-dsDNA positivity, male sex and age of SLE onset on the occurrence of LN. These factors were therefore used for incorporation of the RIFLE-LN risk prediction model.

**Table 4:** Factors associated with lupus nephritis.

	Univariate		Multivariate	
	HR (95% CI)	p-value	HR (95% CI)	p-value
**Male sex**	**1.45 (1.16-1.81)**	**0.001**	**1.40 (1.12-1.75)**	**0.003**
Age of SLE onset (years)				
<18	**1.53 (1.30-1.79)**	**<0.001**	**1.50 (1.09-2.06)**	**0.013**
18-50	**0.74 (0.64-0.86)**	**<0.001**	1.04 (0.77-1.40)	0.797
>50	0.86 (0.64-1.16)	0.321		
Auto-antibodies				
Anti-dsDNA	**1.63 (1.35-1.97)**	**<0.001**	**1.57 (1.30-1.90)**	**<0.001**
Anti-Ro	0.93 (0.78-1.10)	0.383		
Anti-RNP	0.93 (0.76-1.14)	0.482		
Anti-La	0.82 (0.63-1.08)	0.158		
Anti-Sm	0.85 (0.64-1.13)	0.261		
Anti-phospholipid	1.04 (0.88-1.24)	0.643		
Major organ involvement				
Hematological	**1.16 (1.01-1.33)**	**0.041**	1.08 (0.94-1.24)	0.291
Neuropsychiatric	1.05 (0.86-1.27)	0.659		
Pulmonary	**0.78 (0.62-0.98)**	**0.03**	**0.78 (0.62-0.98)**	**0.035**
Cardiac	1.20 (0.88-1.63)	0.243		
Gastrointestinal	0.85 (0.60-1.20)	0.346		

CI, confidence interval; HR, hazard ratio; SLE, systemic lupus erythematosus.Bold values refer to statistical significant results.

### A prediction model for 10-year LN risk in patients with SLE was developed based on four factors: age of SLE onset, male sex, anti-dsDNA autoantibody, and SLE duration

**Figure 3:**
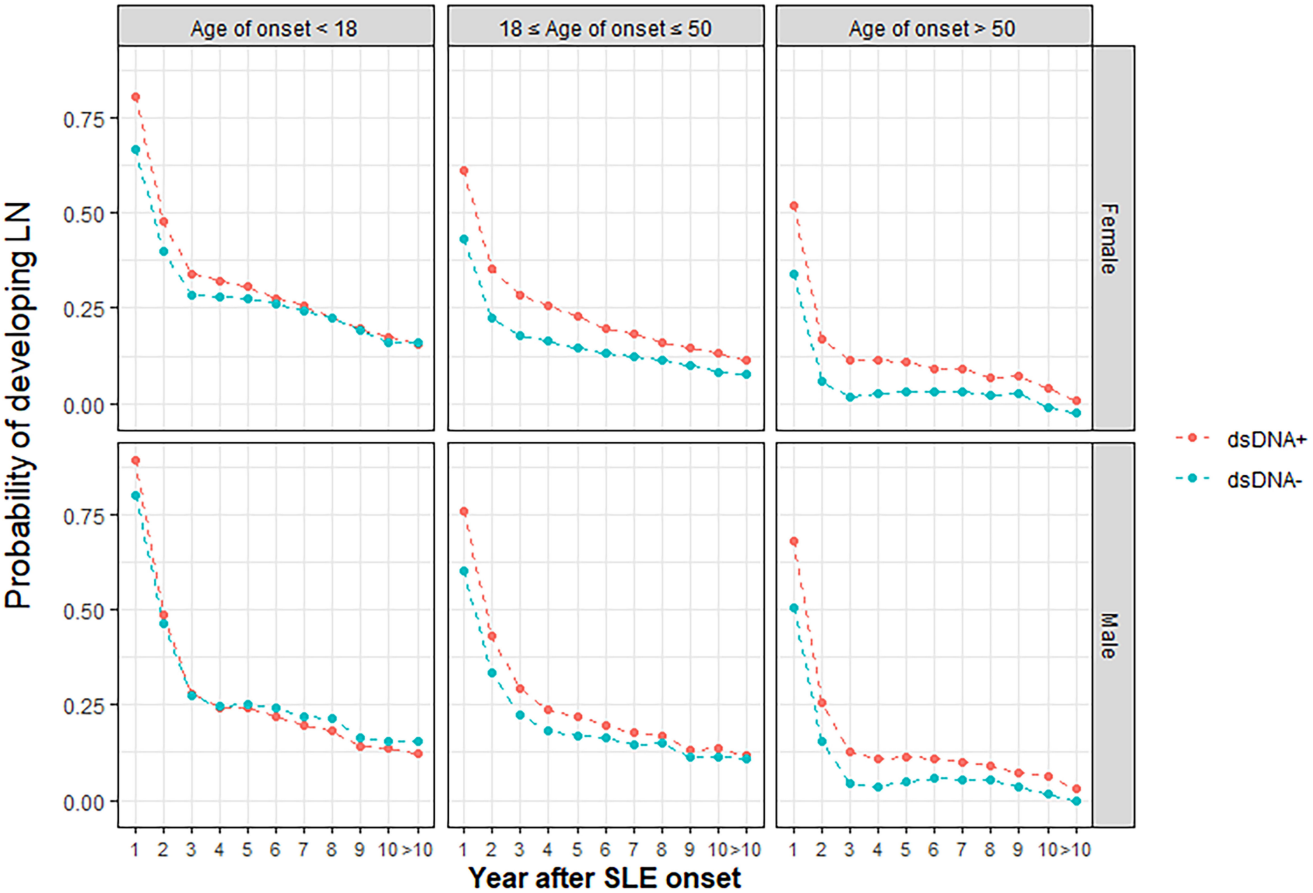
Graphical RIFLE-LN risk prediction model for LN development.

Risk factors identified in regression analysis (age of onset, male sex, and anti-dsDNA autoantibody) and SLE duration were used to develop RIFLE-LN, a prediction model for the 10-year risk of LN development. Model training and validation was repeated ten times, and model parameters with the greatest sensitivity and specificity after ten cycles were used. Probability graphs of RIFLE-LN were generated for visualisation based on age of SLE onset (< 18 years old, 18-50 years old, > 50 years old), sex, anti-dsDNA positivity and SLE duration (**Figure 3**).

### Evaluation in a testing cohort of 270 independent patients demonstrated good performance

**Figure 4:**
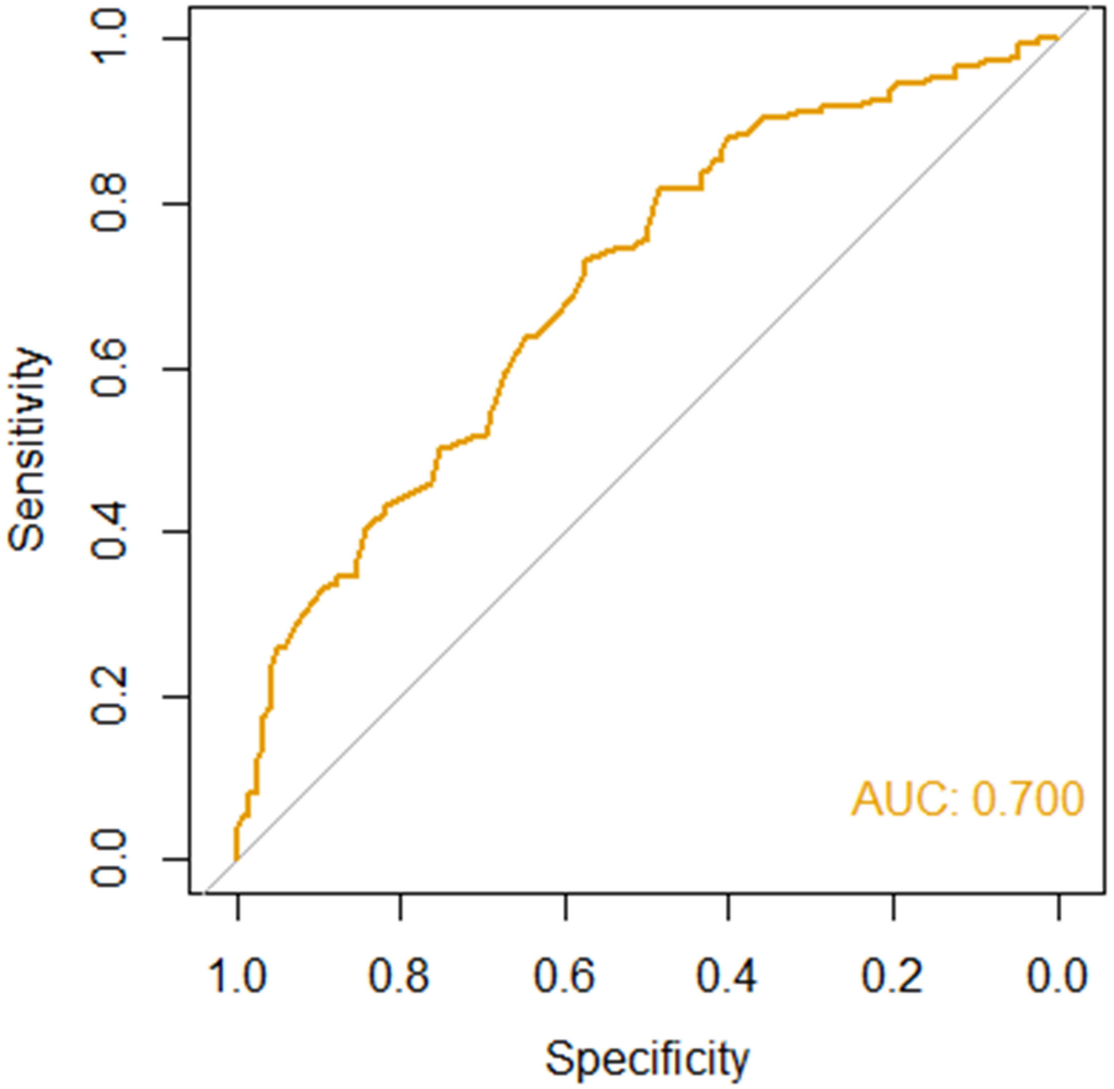
Performance of RIFLE-LN in the testing cohort of 270 independent patients.

The performance of RIFLE-LN was evaluated in a testing cohort of 270 independent patients with SLE. The model performance was assessed using AUC-ROC, and it demonstrated good performance (AUC = 0·70) (**Figure 4**). The sensitivity and specificity were 0·73 and 0·57, respectively.

## Discussion

Using the comprehensive data from a territory-wide longitudinal cohort, we developed a prediction model for the 10-year risk of LN in Chinese patients with SLE. The model demonstrated good performance in LN prediction and a potential role in clinical application.

LN remains a major risk factor for morbidity and mortality in patients with SLE (18). A clinical challenge exists in predicting and identifying patients at risk of LN. The unmet needs are particularly important among Asian SLE patients, who have a higher frequency and more severe LN (19). In our study, the burden of LN was significant; over 60% of patients were diagnosed with LN and 63.6%% had diffuse proliferative LN (or mixed proliferative and membranous). Intriguingly, while some SLE patients experience recurrent LN flares, a substantial proportion of patients do not develop LN during their entire disease course; which suggests marked heterogeneity and the possibility of sub-phenotypes even among LN patients. LN represents a distinct subtype which occurs in certain SLE patients. LN risk prediction among SLE patients may facilitate a more personalised approach in disease monitoring and management; where patients deemed at higher risk of developing LN may benefit from more intensive monitoring, have lower threshold for decision towards invasive renal biopsies or costly immunosuppressive therapies.

The risk of LN development is attributed by a combination of clinical and genetic factors (6). RIFLE-LN is a prediction model developed based on four readily-available features derived from regression analysis. These factors are in line with previous studies as key to LN development: age of SLE onset, male sex, anti-dsDNA autoantibody and SLE duration. Young age of SLE onset has been consistently reported as an important risk factor of LN, likely due to a stronger genetic contribution in disease pathogenesis among these patients (20). The association between male sex and renal involvement highlights the complex interaction of multiple sex hormones involved in SLE (21). On the autoantibody level, loss of immune tolerance and the production of autoantibodies are central to SLE pathogenesis. Anti-dsDNA autoantibody has been strongly linked to LN, and different mechanisms underpinning its nephritogenic potential have been proposed (22). Utilizing age of SLE onset, male sex and anti-dsDNA autoantibody positivity, RIFLE-LN incorporated the different aspects of disease pathogenesis in LN prediction.

Our longitudinal data allowed us to capture a key factor in LN prediction, namely the SLE disease duration. We confirmed that LN often developed early, and 71% of LN in our study occurred within five years of SLE diagnosis. The immunopathogenic mechanisms responsible for SLE initiation, may have directly contributed to LN development. However, LN can develop late in the disease course. Previous studies showed no difference in disease profile and treatment outcome in patients with late-onset LN (23). Maintaining vigilance in identifying LN, especially in patients at ongoing risk is therefore important. RIFLE-LN is a prediction model of 10-year LN risk in SLE. Our algorithm captures the LN probability from each of the first 10 years after SLE diagnosis, addressing the unmet needs of personalised risk assessment and monitoring for LN in SLE.

RIFLE-LN was a multidisciplinary collaboration and developed jointly by a group of rheumatologists, nephrologists, paediatricians, immunologist and bioinformaticians across multiple tertiary centres. Our unique cohort captured a wide spectrum of SLE patients from different specialty clinics. We included over 1000 SLE patients for algorithm training and validation, and comprising different patient subgroups (8·4% patients were male, 18·2% patients had young-onset SLE defined as age of SLE onset younger than 18 years, and 61·1% had LN). Derived from a cohort with diverse disease heterogeneity, RIFLE-LN can be applied across different clinical settings.

Artificial intelligence prediction models have been increasingly applied in medicine and healthcare (24). Regression analysis is one of the commonest prediction models, with the advantages of simple implementation and interpretation. Despite using only four factors, RIFLE-LN demonstrated good performance in our testing cohort. The rapid development in artificial intelligence has changed the landscape of medical research and healthcare in areas including basic research, translational medicine and clinical practice (25). RIFLE-LN aims to improve risk stratification, guide disease monitoring, facilitate early recognition of LN, and complement physician’s judgement in clinical decision and diagnosis.

The exact aetiology of SLE remains elusive, and a combination of polygenic and environmental risk factors are believed to orchestrate disease development. Our centre was among the first to conduct genome-wide association studies (GWAS) of SLE in Asia. Over a hundred of genetic loci have been identified through GWAS analysis (26, 27), and polygenic risk score has been explored as an approach to evaluate an individual’s genetic predisposition from genome-wide risk measurement and the aggregated risk from different disease alleles. Furthermore, the variety of subphenotypes is most likely due to different underlying disease mechanisms contributed by distinct genetic predispositions (28). RIFLE-LN sets a good foundation for LN risk prediction, and we propose that future prediction model should incorporate genetic risk measurement together with clinical and serological features in SLE.

There were several limitations in this study. Despite our large patient cohort, only patients of Chinese ethnicity were included. External validation of the algorithm in patients with other ethnicities is warranted. All patients with LN, with or without renal biopsy, were included. Among patients without renal biopsy, LN was diagnosed by treating physicians based on clinical and laboratory evidence of disease activity and renal involvement. Patient’s refusal, unstable medical conditions, and bleeding risk were the three major reasons why biopsy was not performed. Histological information and classes were not available in patients who did not undergo renal biopsy. However, this approach allowed a more accurate reflection of the true prevalence of LN among patients with SLE. Furthermore, our study the presence of SLE-related autoantibodies was defined as ever-positive, and changes in anti-dsDNA autoantibody titre were not evaluated. RIFLE-LN aims to improve risk stratification of LN but does not replace physician’s judgement in disease diagnosis.

## Conclusion

By using sex, anti-dsDNA positivity, age of onset and SLE duration; RIFLE-LN can predict LN development among Chinese SLE patients with good performance. We advocate its potential utility in guiding patient treatment and disease monitoring. Further validation studies using independent cohorts, especially with different ethnicities and populations, are required.

## Data availability statement

The original contributions presented in the study are included in the article/**Supplementary Material**; further inquiries can be directed to the corresponding authors.

## Ethics statement

The studies involving human participants were reviewed and approved by Hospital Authority Central Institutional Review Board. Written informed consent for participation was not required for this study in accordance with the national legislation and the institutional requirements.

## Author contributions

TC, YL, WL, PHL, and CL contributed to study planning and design. PPL, WL, SY, NT, AL, CM, KL, TL, HHL, WWY, and CH contributed to the acquisition, analysis or interpretation of data. SC, and PHL contributed to drafting of the manuscript. TC, YL, WL, RW, and CL contributed to the critical revision of the manuscript. The first and corresponding authors had full access of all the data in the study and had final responsibility for the decision to submit the manuscript for publication. All authors contributed to the article and approved the submitted version.
