# Supplementary material for: Risk and Factors associated with disease manifestations in systemic lupus erythematosus – lupus nephritis (RIFLE-LN): a ten-year risk prediction strategy derived from a cohort of 1652 patients
Source: Front Immunol. 2023 Jun 15;14:1200732. doi: 10.3389/fimmu.2023.1200732 (PMC10311203; doi:10.3389/fimmu.2023.1200732)
Supplement: Supplementary file 1 [file Table_1.pdf]

Supplementary Table 1: characteristics of the training and validation cohort and the testing cohort.

	**Training and validation cohort (N=1382)**	**Testing cohort (N=270)**	**P-value**
**Age of SLE onset (median, IQR)**	29 (18)	30 (15)	0.094
**Male sex**	116/ 1382 (8.4%)	27/270 (10.0%)	0.391
**Duration of follow-up (median, IQR)**	21 (11)	19 (15)	0.376
**Auto-antibodies**			
Anti-dsDNA	1096/1382 (79.3%)	218/270 (80.7%)	
Anti-Ro	420/898 (46.8%)	98/195 (50.3%)	0.324
Anti-RNP	201/898 (22.4%)	49/195 (25.1%)	0.402
Anti-La	110/898 (12.2%)	35/195 (17.9%)	**0.039**
Anti-Sm	94/898 (10.5%)	33/195 (16.9%)	**0.015**
Anti-phospholipid	262/1146 (22.9%)	38/151 (25.2%)	0.345
**Major organ involvement**			
Lupus nephritis	845/1382 (61.1%)	148/270 (54.8%)	0.052
Hematological	805/1382 (58.2%)	165/270 (61.1%)	0.359
Neuropsychiatric	187/1382 (13.5%)	25/270 (9.3%)	0.071
Pulmonary	150/1382 (10.9%)	35/370 (13.0%)	0.297
Cardiac	64/1382 (4.6%)	11/270 (4.1%)	0.652
Gastrointestinal	59/1382 (4.3%)	8/270 (3.0%)	0.344
